# Supplementary material for: Three New Compounds, Licopyranol A–C, Together with Eighteen Known Compounds Isolated from Glycyrrhiza glabra L. and Their Antitumor Activities
Source: Metabolites. 2022 Sep 23;12(10):896. doi: 10.3390/metabo12100896 (PMC9612088; doi:10.3390/metabo12100896)
Supplement: Supplementary file 1 [file metabolites-12-00896-s001.zip › metabolites-1880436-supplementary.pdf]

Supplementary materials

List of Contents

Figure S1. The inhibitory effects of the extracts of *Glycyrrhiza glabra* on RKO and HT-29 cells treated with 10, 20, 40, and 80 μg/ml for 48 h.

Figure S2 UV spectrum of compound **1**

Figure S3 HR-ESI-MS spectrum of compound **1**

Figure S4 ^{1}H NMR spectrum of compound **1** in DMSO-d_6 (400 MHz)

Figure S5 ^{13}C NMR spectrum of compound **1** in DMSO-d_6 (100 MHz)

Figure S6 DEPT 135 spectrum of compound **1** in DMSO-d_6

Figure S7 HSQC spectrum of compound **1** in DMSO-d_6

Figure S8 HMBC spectrum of compound **1** in DMSO -d_6

Figure S9 ^{1}H-^{1}H COSY spectrum of compound **1** in DMSO-d_6

Figure S10 UV spectrum of compound **2**

Figure S11 HR-ESI-MS spectrum of compound **2**

Figure S12 ^{1}H NMR spectrum of compound **2** in DMSO-d_6 (400 MHz)

Figure S13 ^{13}C NMR spectrum of compound **2** in DMSO-d_6 (100 MHz)

Figure S14 DEPT 135 spectrum of compound **2** in DMSO-d_6

Figure S15 HSQC spectrum of compound **2** in DMSO-d_6

Figure S16 HMBC spectrum of compound **2** in DMSO-d_6

Figure S17 ^{1}H-^{1}H COSY spectrum of compound **2** in DMSO-d_6

Figure S18 UV spectrum of compound **3**

Figure S19 HR-ESI-MS spectrum of compound **3**

Figure S20 ^{1}H NMR spectrum of compound **3** in DMSO-d_6 (400 MHz)

Figure S21 ^{13}C NMR spectrum of compound **3** in DMSO-d_6 (100 MHz)

Figure S22 DEPT 135 spectrum of compound **3** in DMSO-d_6

Figure S23 HSQC spectrum of compound **3** in DMSO-d_6

Figure S24 HMBC spectrum of compound **3** in DMSO-d_6

Figure S25 ^{1}H-^{1}H COSY spectrum of compound **3** in DMSO-d_6

Table S1 Rf value and UV lambda Max of Compounds **4-21**

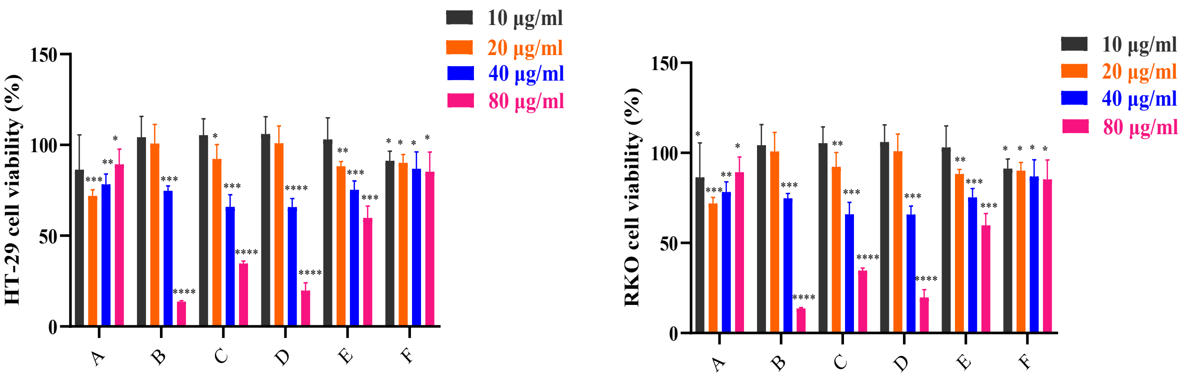

Figure S1: The effects of the extracts of *Glycyrrhiza glabra* on RKO and HT-29 cells treated with 10, 20, 40 and 80μg/ml for 48 h; *P < 0.05, **P < 0.01, ***P < 0.001, ****P < 0.0001, compared with corresponding control groups (A: 70% MeOH extract of G. glabra, B: Petroleum ether extract, C: Dichloromethane extract, D: Ethyl acetate extract, E: *n*-butanol extract, F: Water extract)

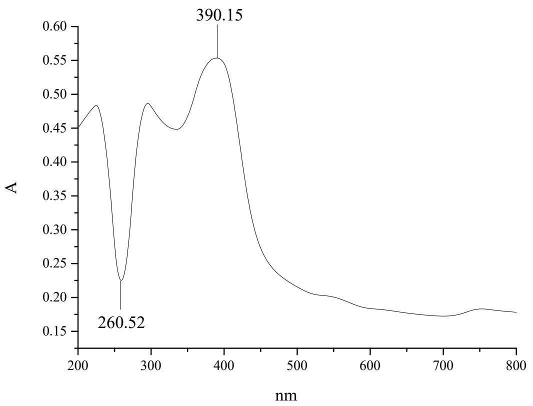

Figure S2. UV spectrum of compound **1**

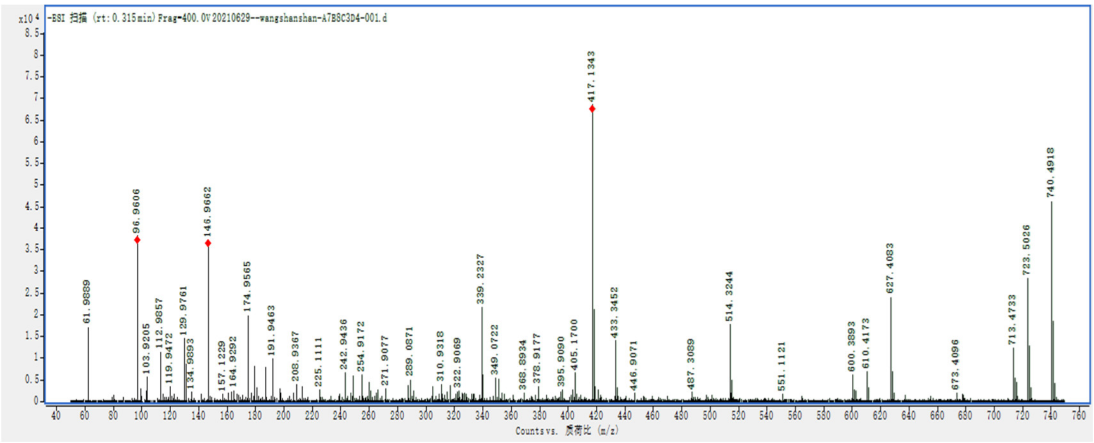

Figure S3. HR-ESI-MS spectrum of compound **1**

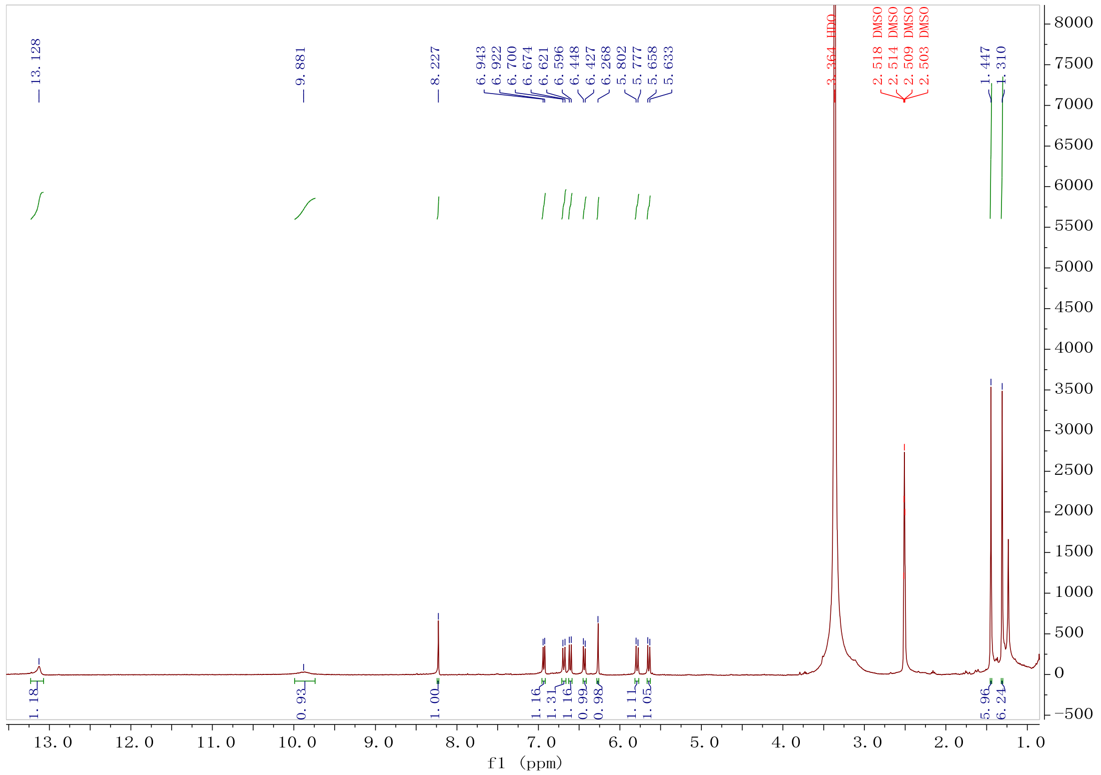

Figure S4. ^{1}H NMR spectrum of compound **1** in DMSO-d_6 (400 MHz)

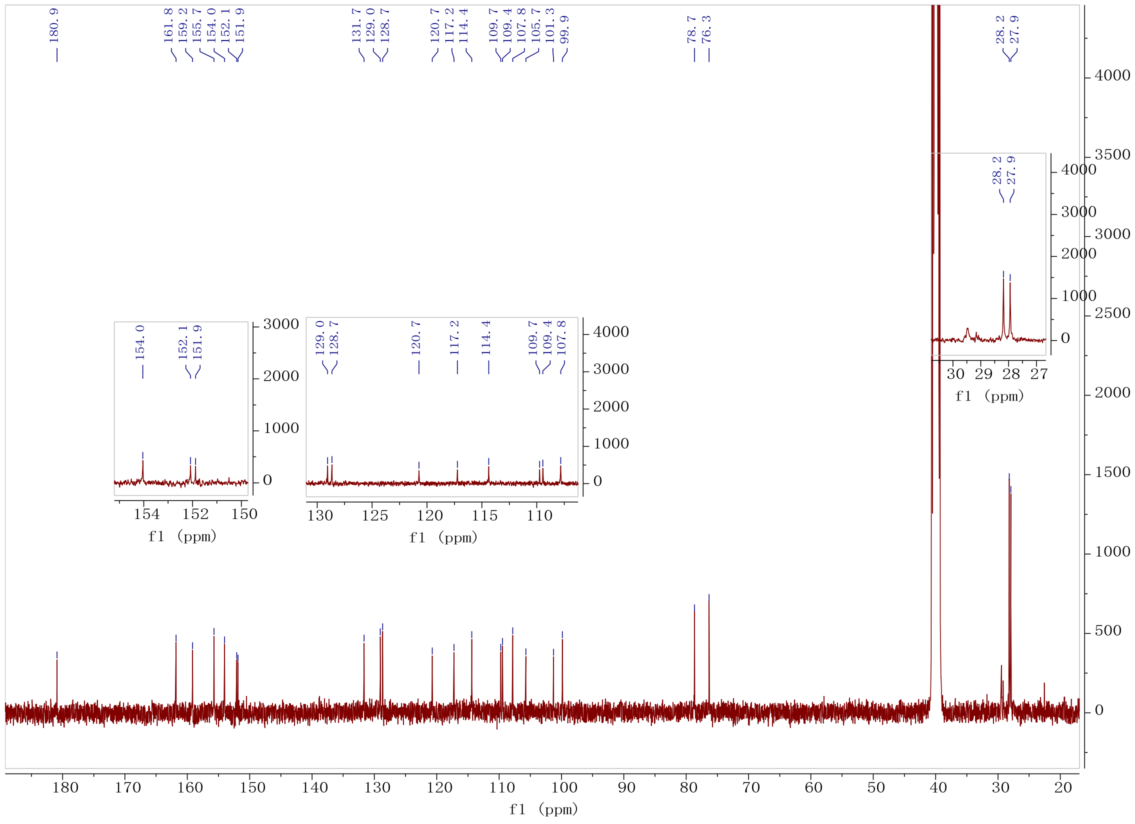

Figure S5. ^{13}C NMR spectrum of compound **1** in DMSO-d_6 (100 MHz)

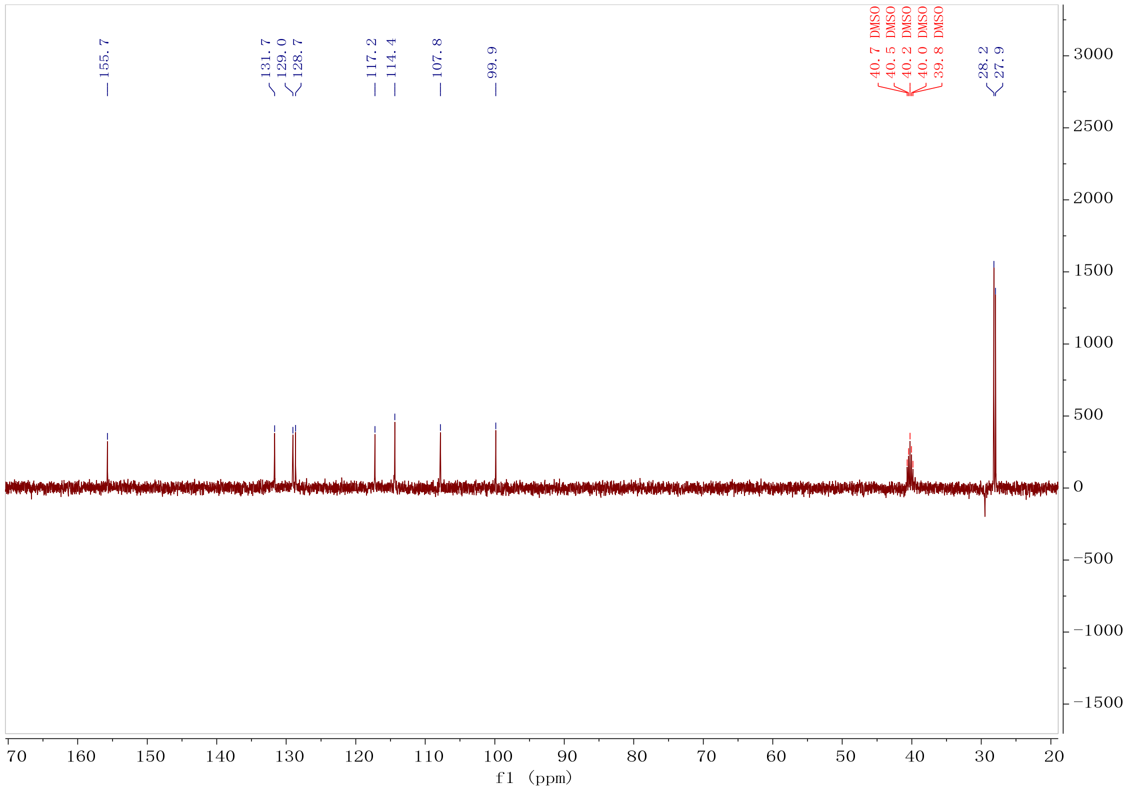

Figure S6. DEPT 135 spectrum of compound **1** in DMSO-d_6

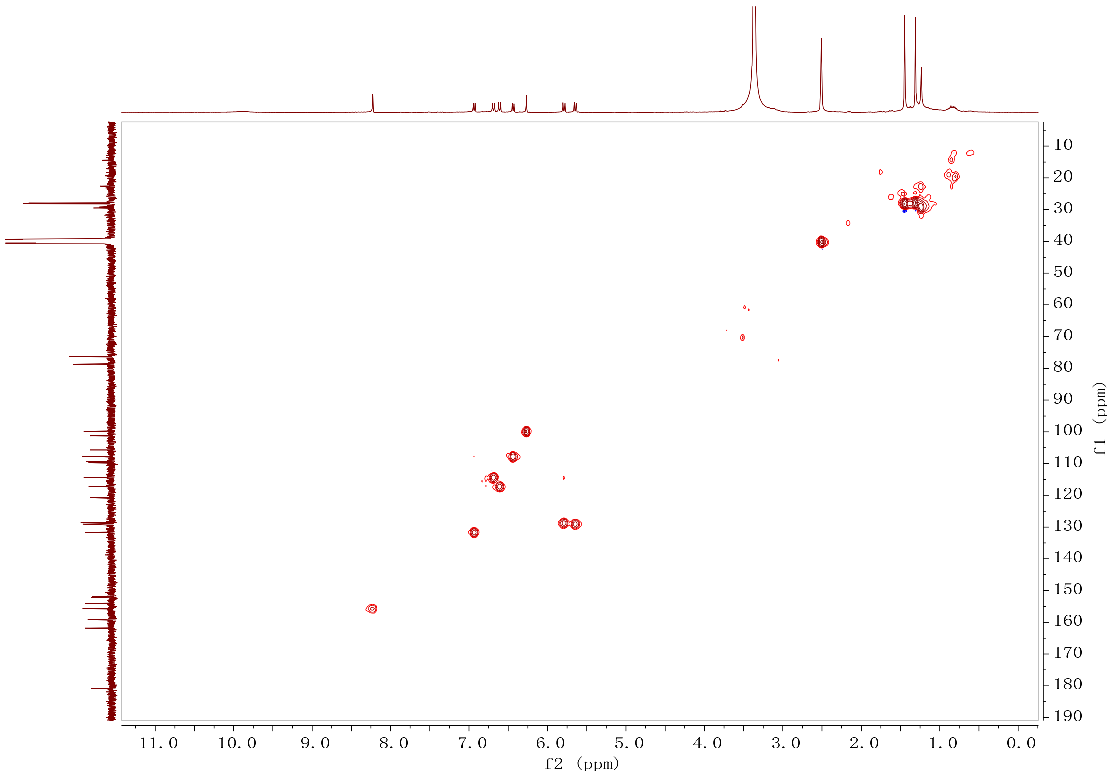

Figure S7. HSQC spectrum of compound **1** in DMSO-d_6

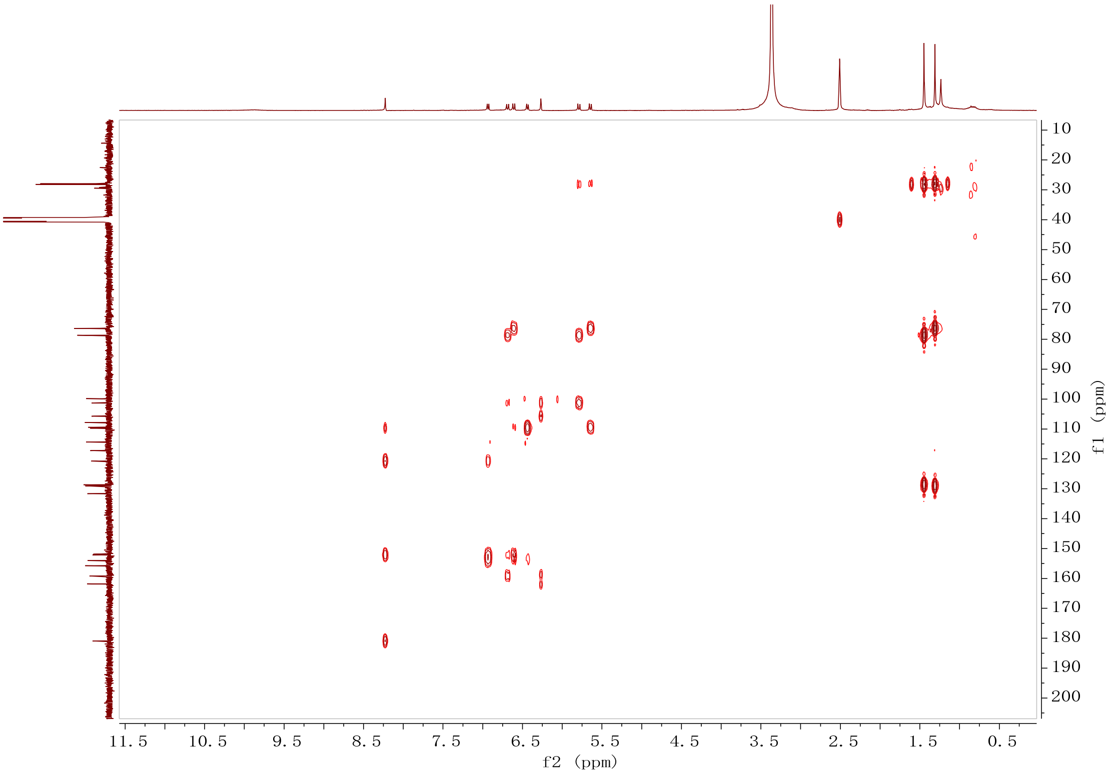

Figure S8. HMBC spectrum of compound **1** in DMSO -d_6

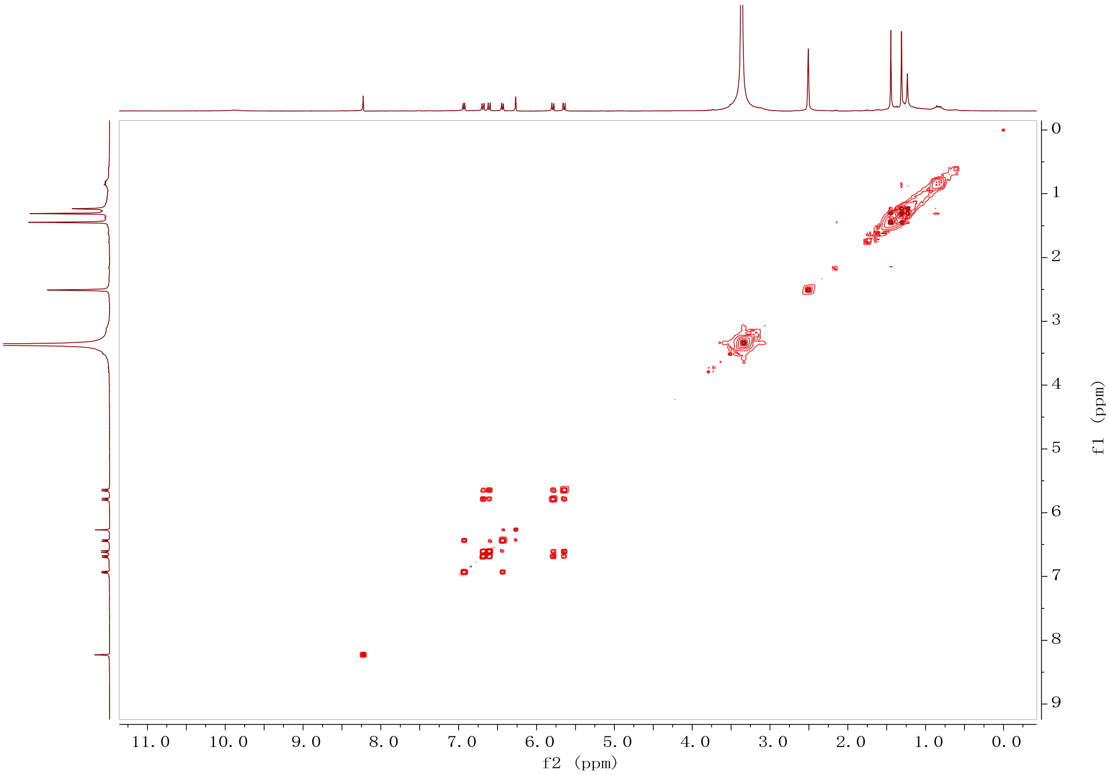

Figure S9. ^1H-^1H COSY spectrum of compound **1** in DMSO-d_6

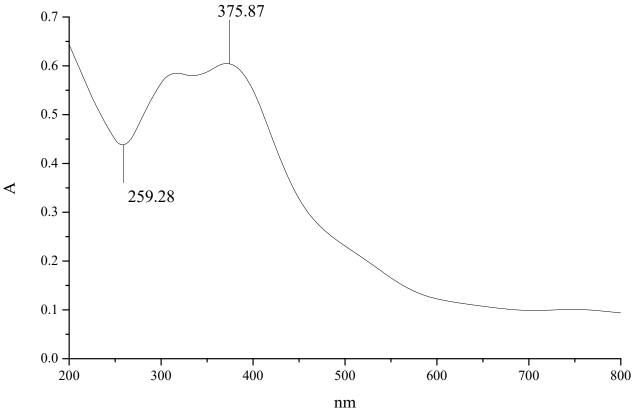

Figure S10. UV spectrum of compound **2**

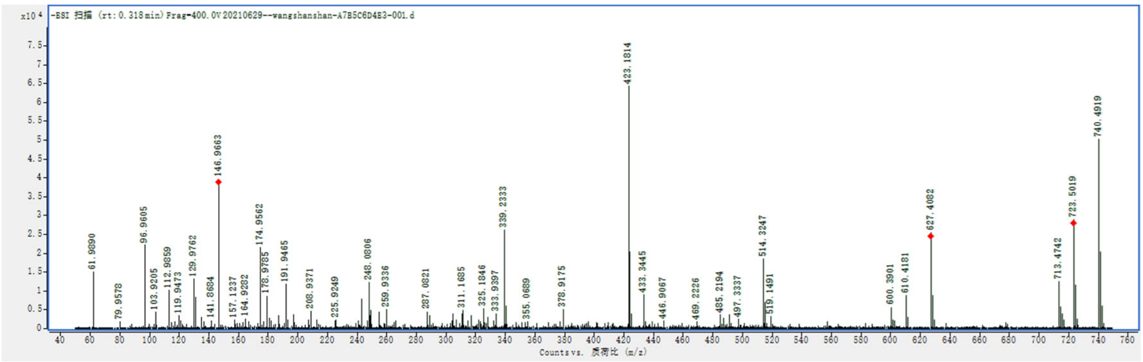

Figure S11. HR-ESI-MS spectrum of compound **2**

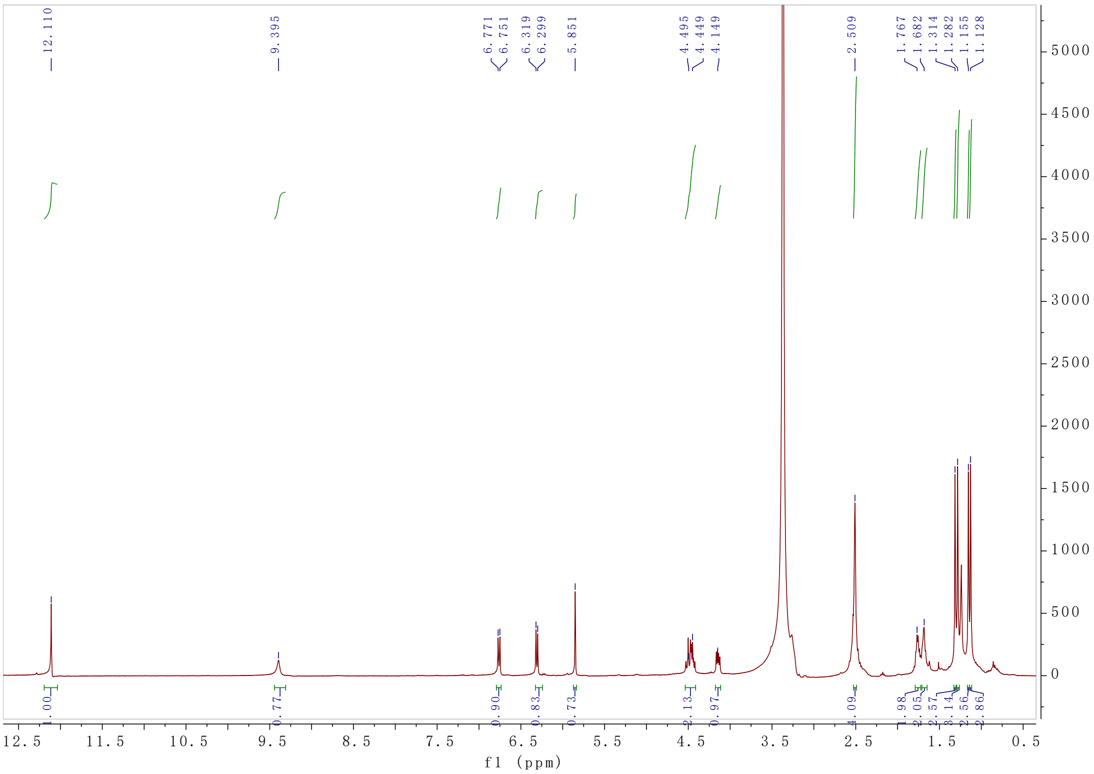

Figure S12. ^{1}H NMR spectrum of compound **2** in DMSO-d_6 (400 MHz)

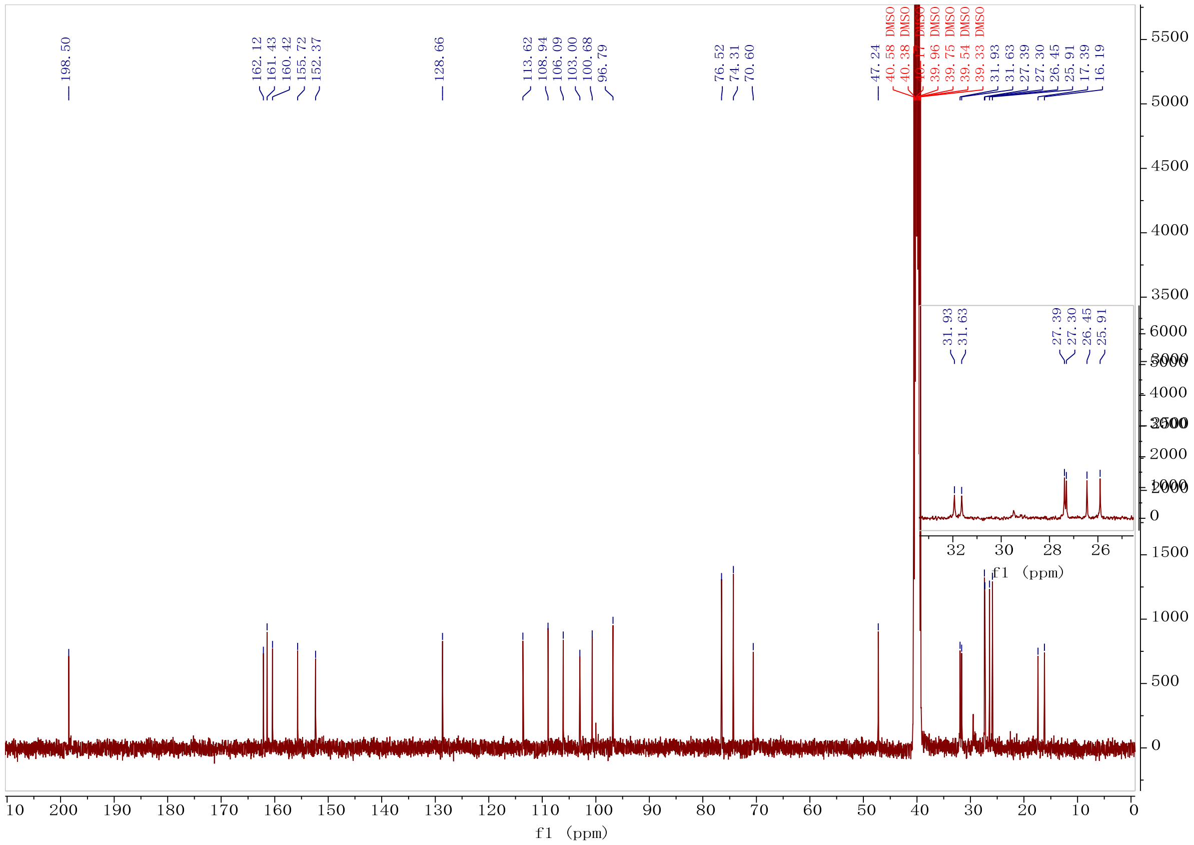

Figure S13. ^{13}C NMR spectrum of compound **2** in DMSO-d_6 (100 MHz)

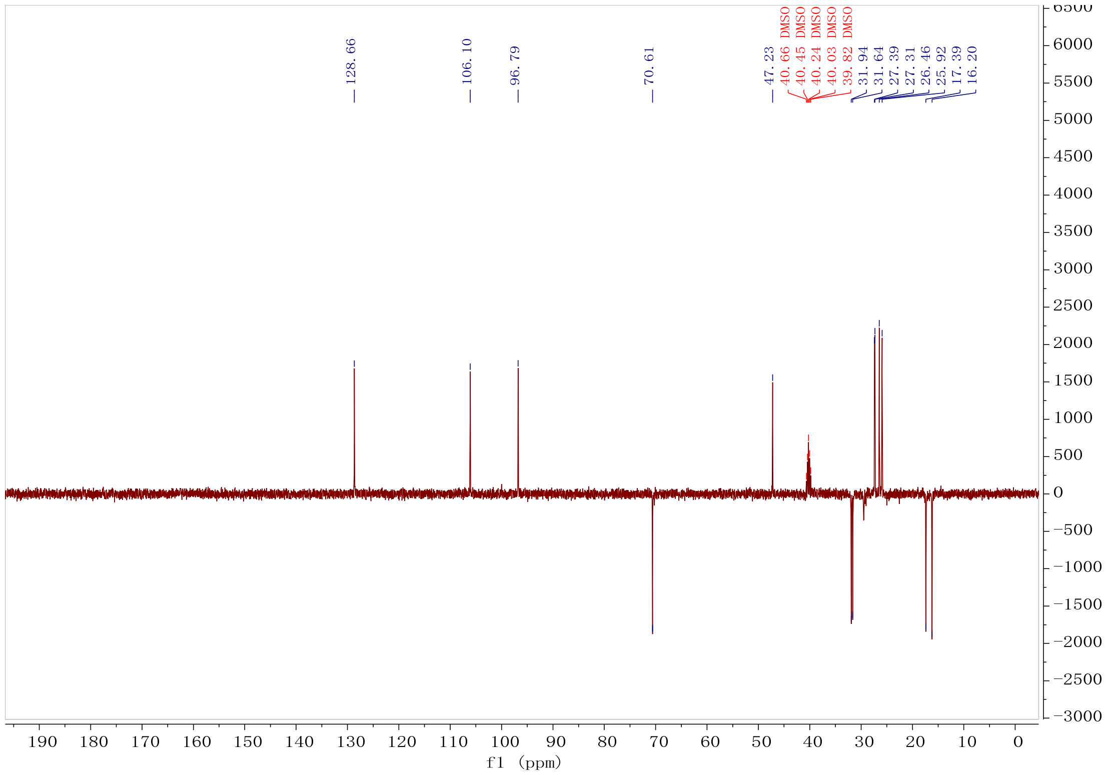

Figure S14. DEPT 135 spectrum of compound **2** in DMSO-*d*

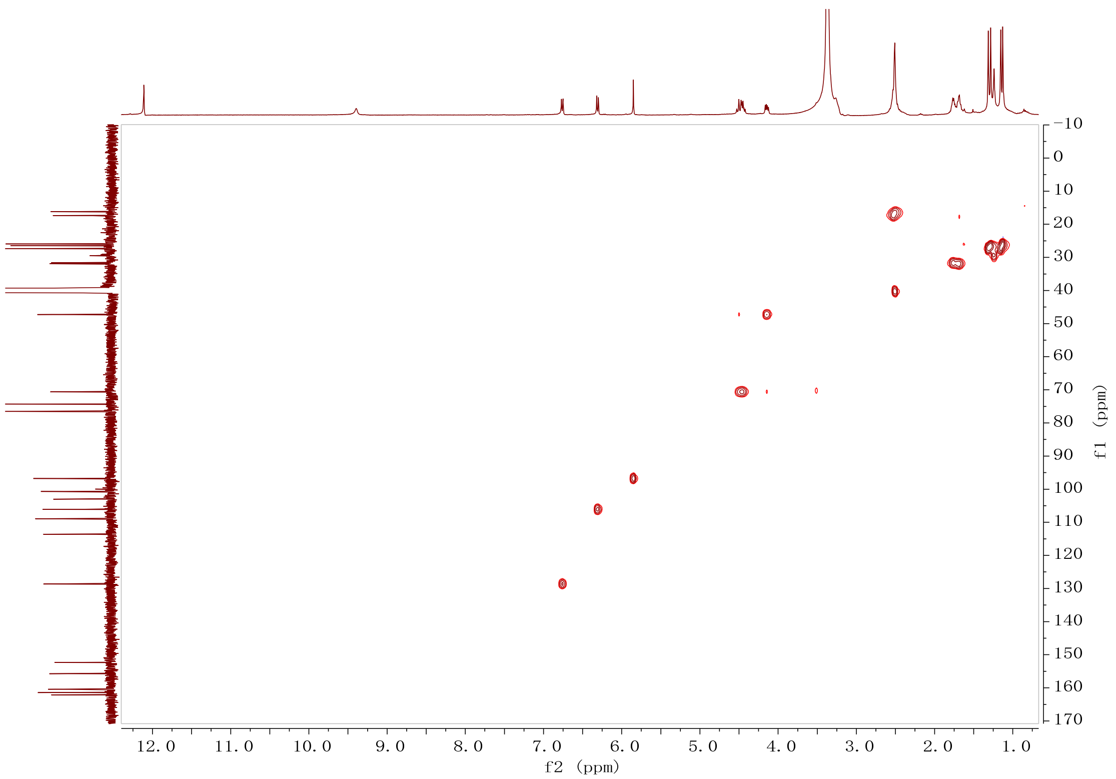

Figure S15. HSQC spectrum of compound **2** in DMSO-d_6

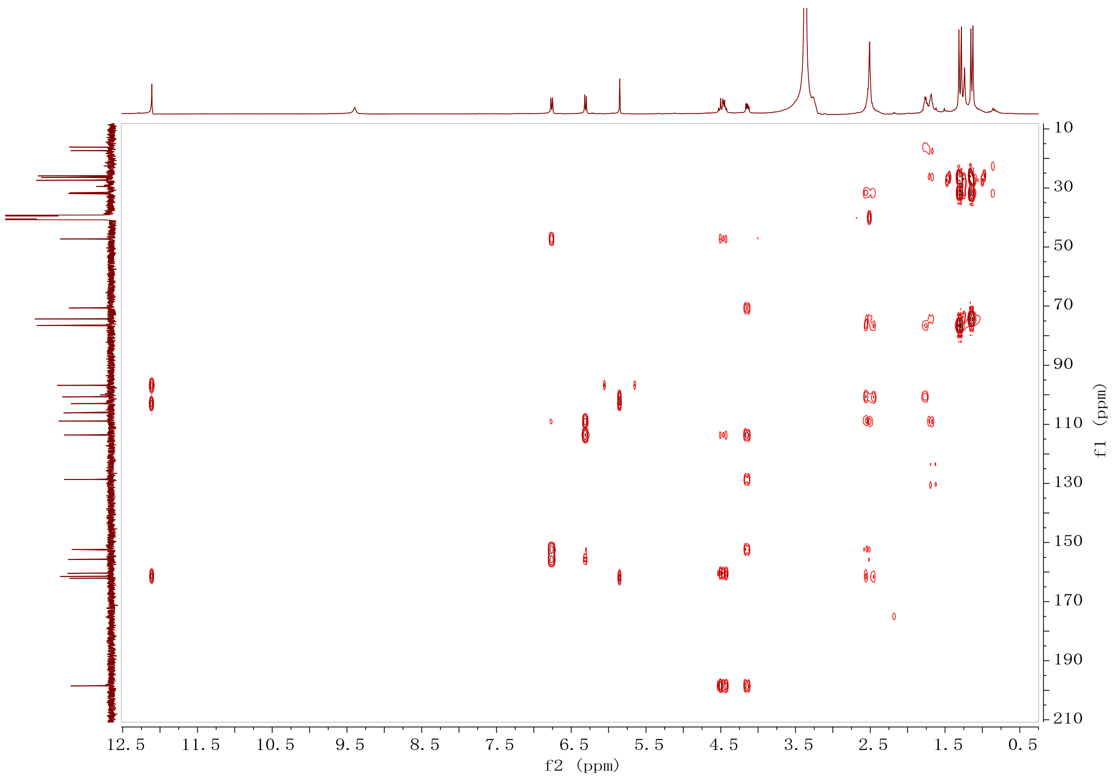

Figure S16. HMBC spectrum of compound **2** in DMSO-d_6

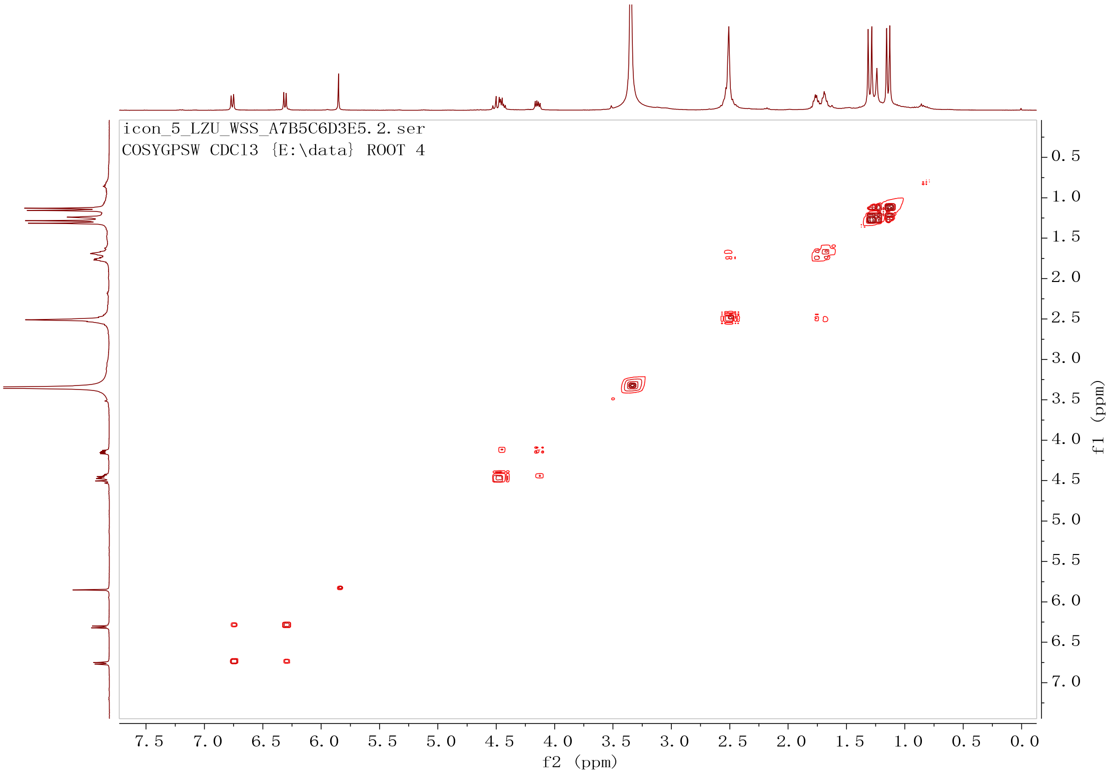

Figure S17. ^{1}H-^{1}H COSY spectrum of compound **2** in DMSO-d_6

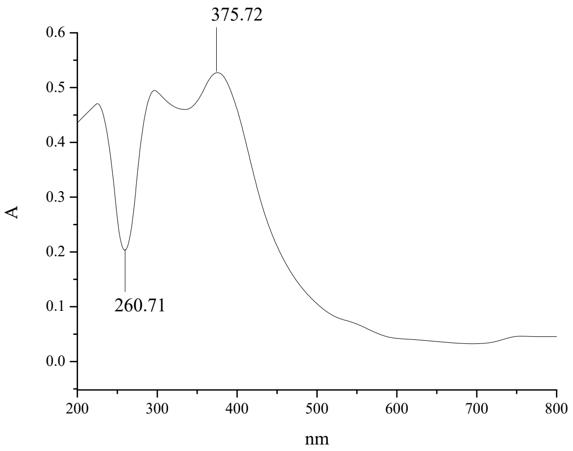

Figure S18. UV spectrum of compound **3**

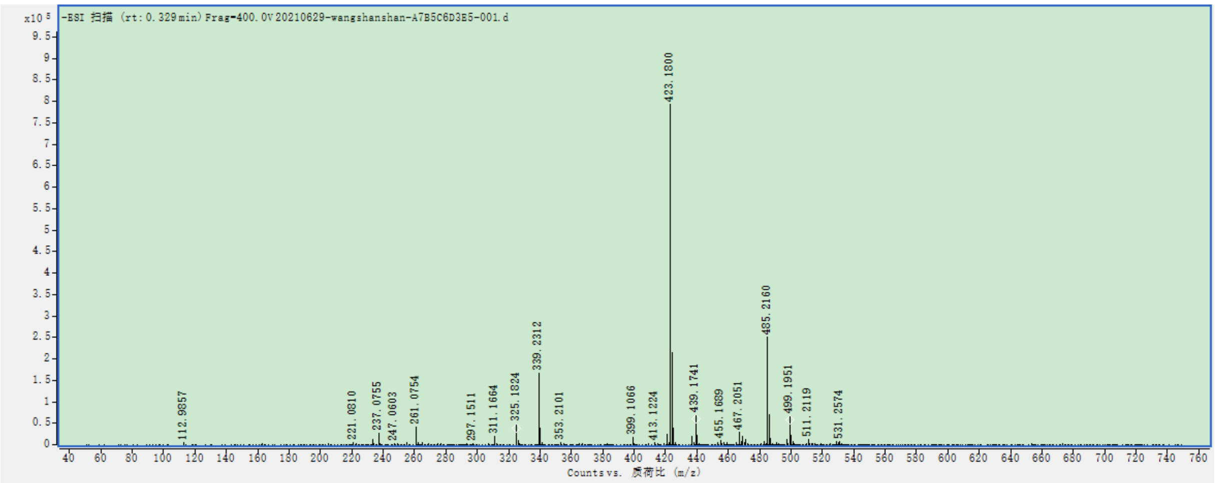

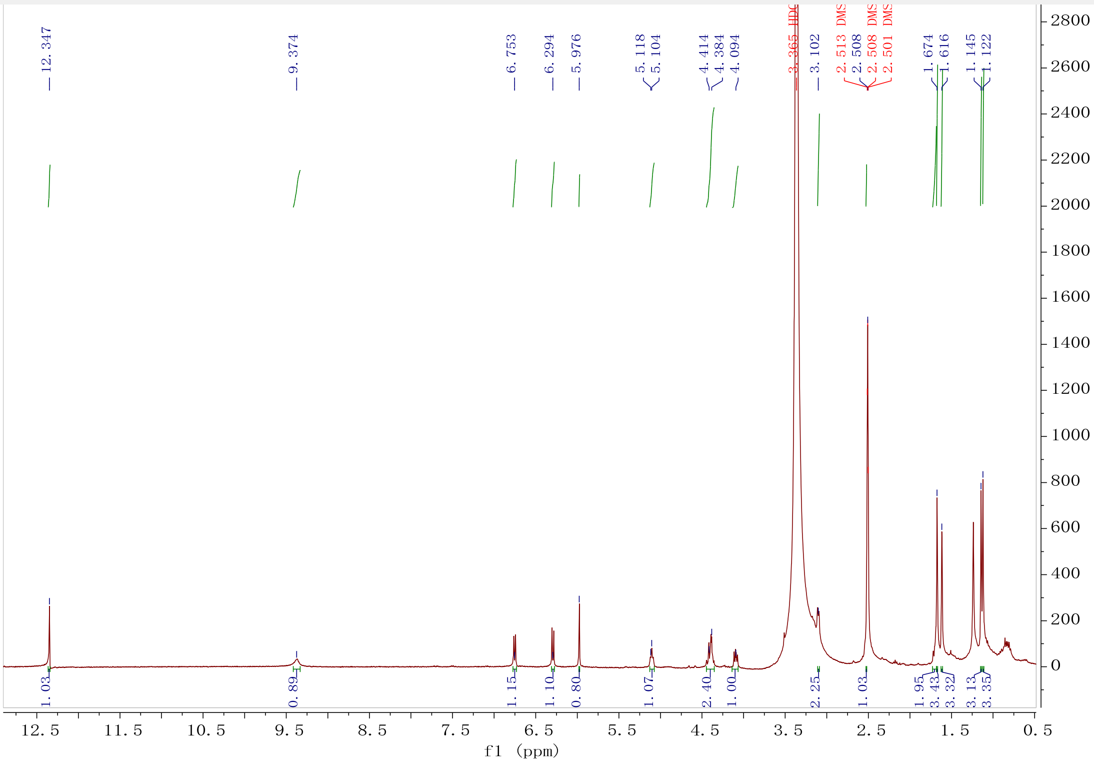

Figure S20. ^{1}H NMR spectrum of compound **3** in DMSO-d_6 (400 MHz)

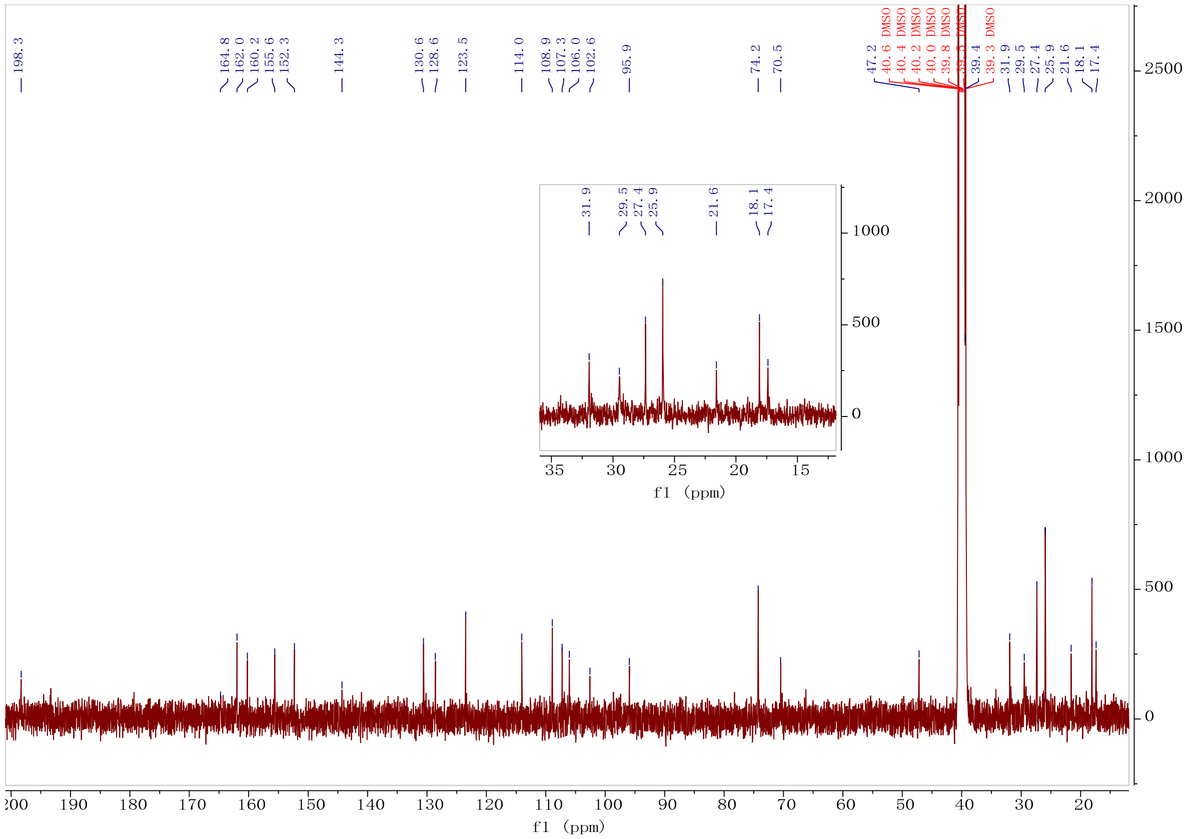

Figure S21. ^{13}C NMR spectrum of compound **3** in DMSO-d_6 (100 MHz)

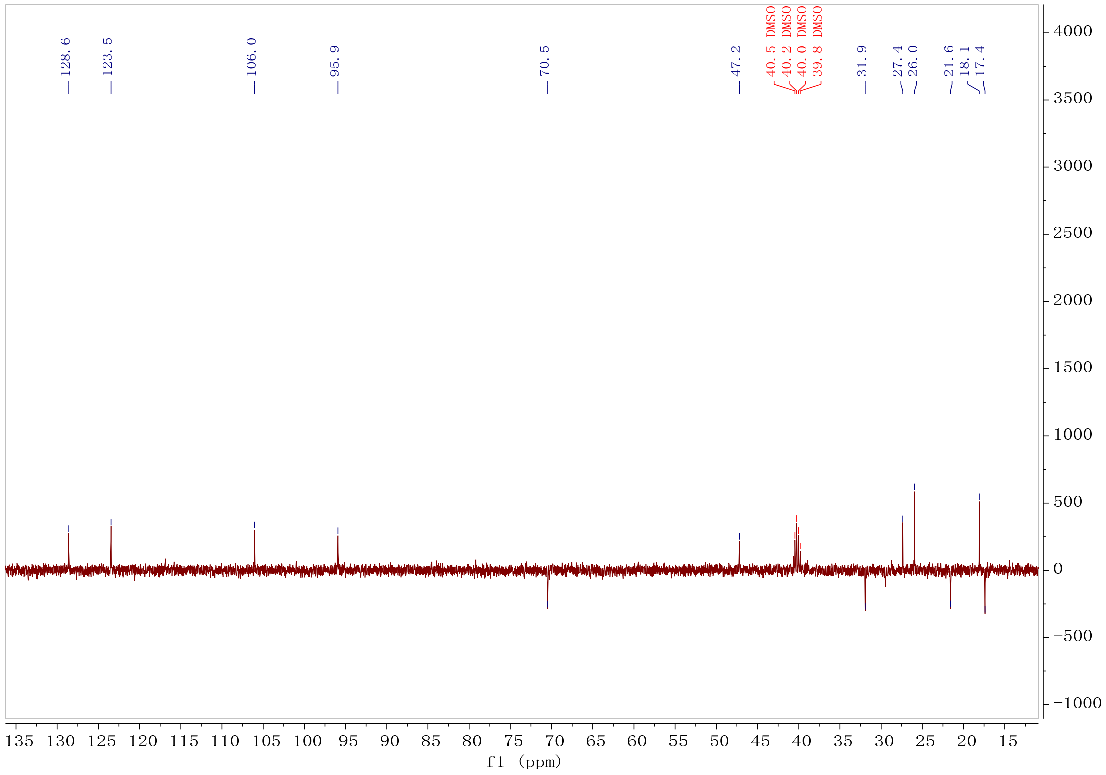

Figure S22. DEPT 135 spectrum of compound **3** in DMSO-d_6

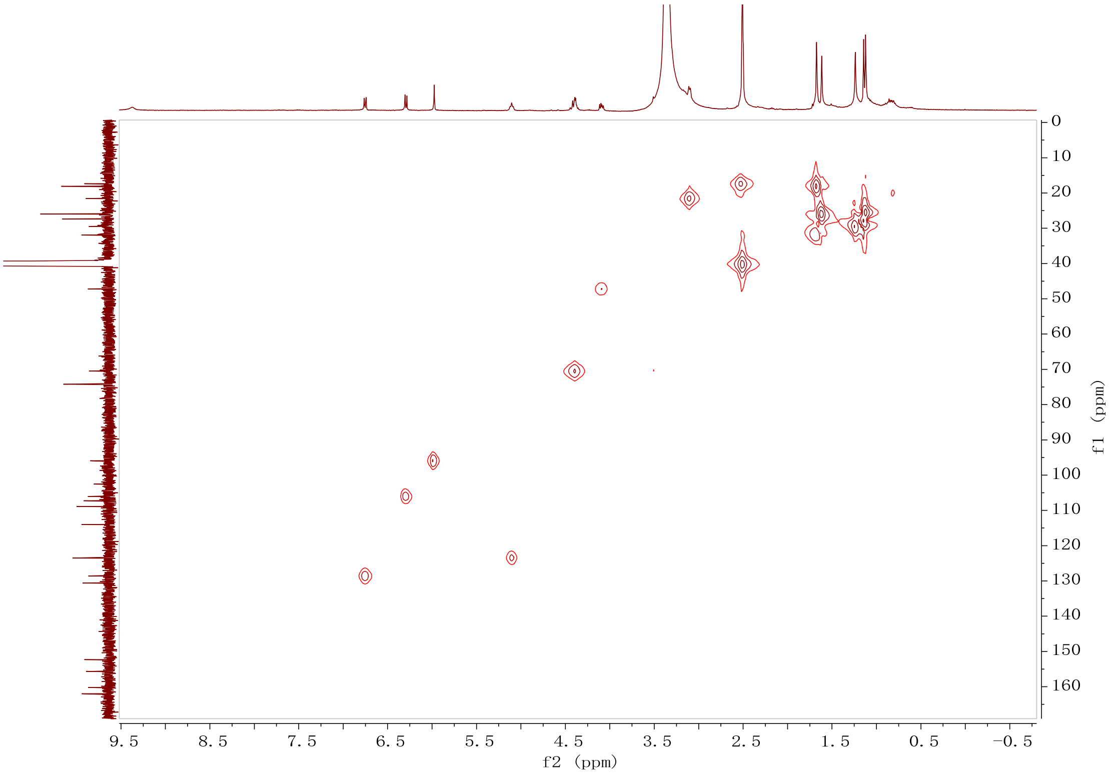

Figure S23. HSQC spectrum of compound **3** in DMSO-d_6

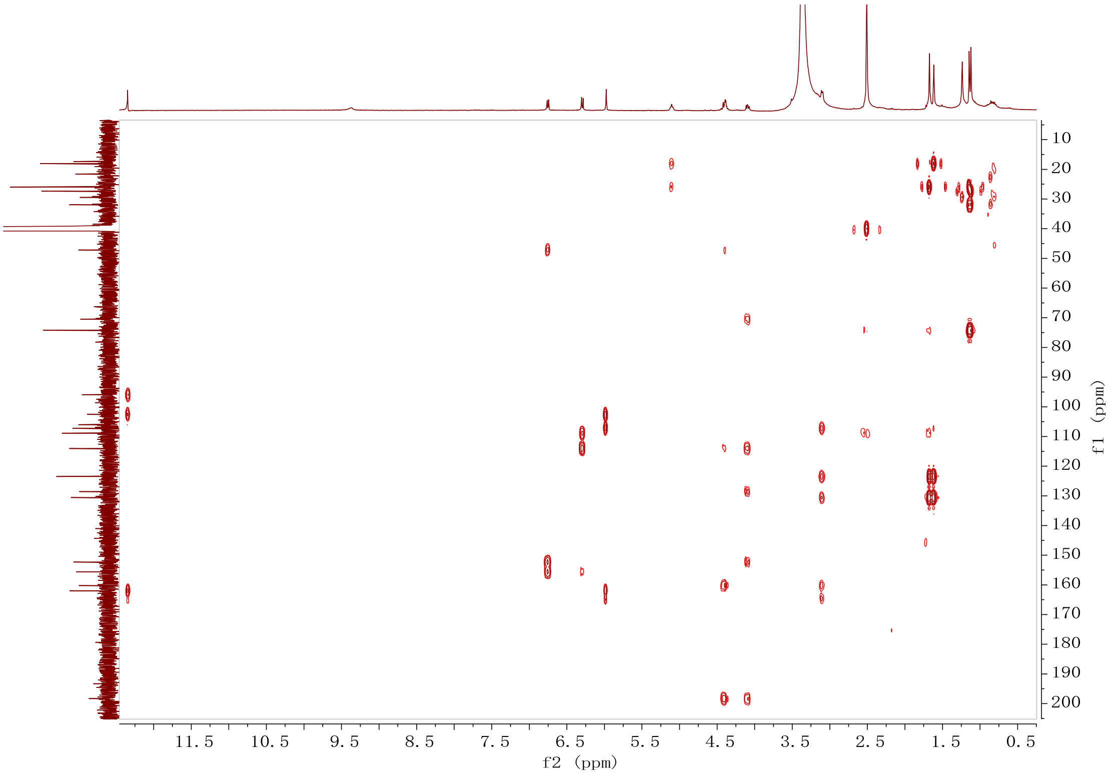

Figure S24. HMBC spectrum of compound **3** in DMSO-d_6

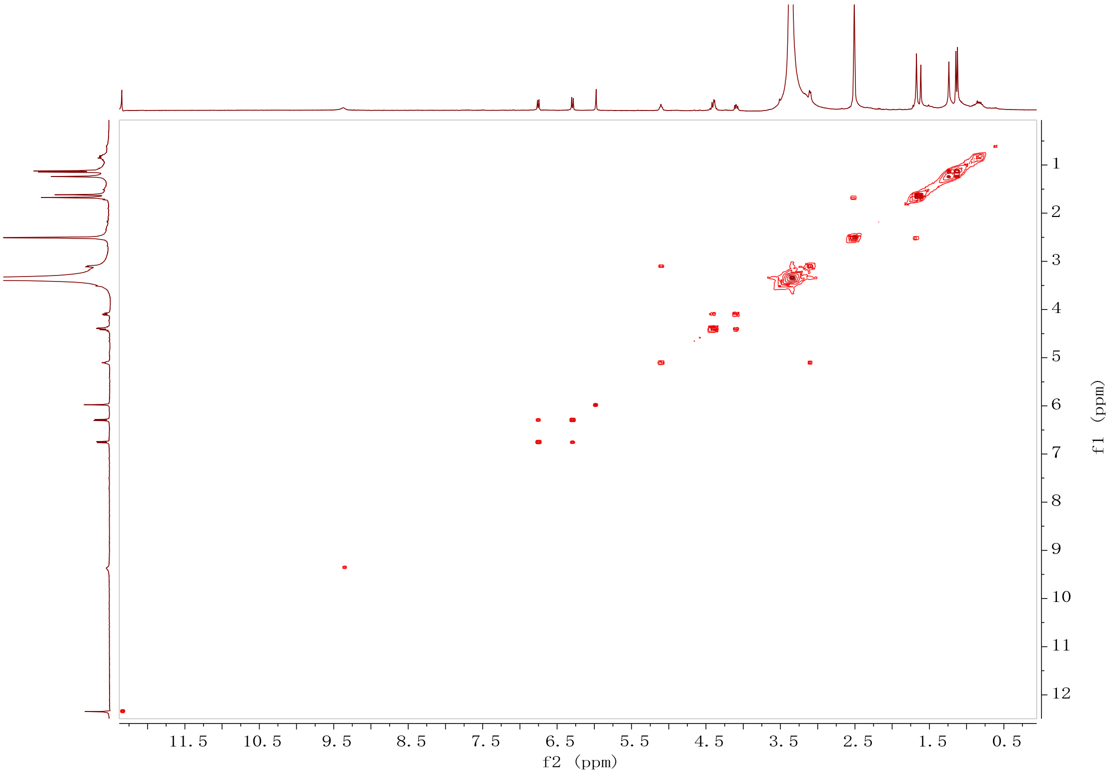

Figure S25. ^1H-^1H COSY spectrum of compound **3** in DMSO-d_6

Table S1. Rf value and UV lambda Max of Compounds **4-21.**

	**$CHCl_3$: MeOH(15:1) Rf**	**CH_2Cl_2 : MeOH(20:1) Rf**	**Petroleum ether: acetone (7.5:2.5) Rf**	**Wavelength (nm)**
**4**	0.67	0.51	0.68	233, 324
**5**	0.49	0.34	0.23	231, 276, 373
**6**	0.34	0.25	0.62	226 ,228 ,351
**7**	0.71	0.54	0.45	271
**8**	0.49	0.31	0.31	268
**9**	0.23	0.22	0.51	208, 264
**10**	0.40	0.25	0.22	209, 265
**11**	0.43	0.23	0.20	208 ,267
**12**	0.60	0.37	0.34	121 ,269
**13**	0.91	0.80	0.54	220 ,267
**14**	0.68	0.46	0.45	218 ,260
**15**	0.80	0.28	0.25	212, 261
**16**	0.60	0.37	0.28	210, 262
**17**	0.65	0.45	0.37	206, 295
**18**	0.45	0.29	0.20	233, 279, 369
**19**	0.66	0.40	0.31	222, 289
**20**	0.69	0.48	0.34	217 ,279 ,318
**21**	0.80	0.57	0.40	229, 299
